# Created mangrove wetlands store belowground carbon and surface elevation change enables them to adjust to sea-level rise

**DOI:** 10.1038/s41598-017-01224-2

**Published:** 2017-04-21

**Authors:** Ken W. Krauss, Nicole Cormier, Michael J. Osland, Matthew L. Kirwan, Camille L. Stagg, Janet A. Nestlerode, Marc J. Russell, Andrew S. From, Amanda C. Spivak, Darrin D. Dantin, James E. Harvey, Alejandro E. Almario

**Affiliations:** 1 U.S. Geological Survey, Wetland and Aquatic Research Center, 700 Cajundome Blvd., Lafayette, Louisiana 70506 USA; 2 Virginia Institute of Marine Science, College of William and Mary, Gloucester Point, Virginia 23062 USA; 3 U.S. Environmental Protection Agency, Gulf Ecology Division, 1 Sabine Island Drive, Gulf Breeze, Florida 32561 USA; 4 Woods Hole Oceanographic Institution, Marine Chemistry and Geochemistry, 266 Woods Hole Rd., Woods Hole, Massachusetts 02543 USA

## Abstract

Mangrove wetlands provide ecosystem services for millions of people, most prominently by providing storm protection, food and fodder. Mangrove wetlands are also valuable ecosystems for promoting carbon (C) sequestration and storage. However, loss of mangrove wetlands and these ecosystem services are a global concern, prompting the restoration and creation of mangrove wetlands as a potential solution. Here, we investigate soil surface elevation change, and its components, in created mangrove wetlands over a 25 year developmental gradient. All created mangrove wetlands were exceeding current relative sea-level rise rates (2.6 mm yr^−1^), with surface elevation change of 4.2–11.0 mm yr^−1^ compared with 1.5–7.2 mm yr^−1^ for nearby reference mangroves. While mangrove wetlands store C persistently in roots/soils, storage capacity is most valuable if maintained with future sea-level rise. Through empirical modeling, we discovered that properly designed creation projects may not only yield enhanced C storage, but also can facilitate wetland persistence perennially under current rates of sea-level rise and, for most sites, for over a century with projected medium accelerations in sea-level rise (IPCC RCP 6.0). Only the fastest projected accelerations in sea-level rise (IPCC RCP 8.5) led to widespread submergence and potential loss of stored C for created mangrove wetlands before 2100.

## Introduction

Mangrove wetlands occupy 83,495^1^ to >137,000 km^2^ of coastline^2, 3^, providing ecosystem services for millions of people^4^. Loss of these services is a global concern^5^, including the efficiency with which tidal wetlands sequester carbon (C) as persistent biomass in soils^6–9^. Creation, restoration, and rehabilitation of tidal ecosystems, such as mangrove wetlands, are important components of climate change mitigation strategies for coastal societies globally^5^, in part because of C storage in roots and soils^10, 11^. Mangrove wetlands are also capable of adjusting their soil surface elevations adaptively with sea level to help influence their course along populated coastlines, as long as sea-level rise rates are not too high and the mangrove ecosystem itself remains relatively healthy^12^.

Successful restoration of mangrove wetlands can be undertaken using several techniques, but in southern Florida, USA, often begins by mechanically grading or modifying shorelines to a known intertidal elevation to facilitate natural tidal flooding, followed by planting or relying on natural recruitment of mangrove trees^13^. In some cases, projects might simply involve breaking down physical shoreline barriers to restore tidal connectivity^14^; however, at times more careful consideration of biogeochemical conditions is necessary to ensure successful restoration. In tropical and sub-tropical regions, herbaceous macrophytes, once established, can facilitate the trapping of mangrove propagules where there is a local propagule supply from surrounding forests^15–17^. As mangrove wetlands develop, sediment deposition, leaf and branch litter fall, sediment retention, emergent root structure, belowground root volume expansion and contraction, and organic matter decomposition all contribute to soil surface elevation change^12, 18^. However, created mangrove wetlands must adjust soil surface elevations with sea-level rise, or at least at rates high enough to facilitate community development that coincides with the appropriate time scale of targeted benefits.

Surface elevation change for natural mangrove wetlands ranges from −5.8 to 6.3 mm yr^−1^, a rate that reflects a balance between vertical accretion of sediments (range from 0.7 to 20.8 mm yr^−1^) and sub-surface change from compaction, decomposition, and/or root zone expansion (range from −19.9 to 2.8 mm yr^−1^)^12, 19, 20^. Yet, mangrove wetlands often experience surface elevation deficits (that is, sea-level rise >surface elevation change) as a response to natural geomorphic evolution or as a result of anthropogenic impact. For example, nearly 69% of mangrove wetlands included in a recent meta-analysis from the Indo-Pacific were actively submerging naturally, but with some sites, such as coastal Vietnam, submerging at a higher rate as a consequence of reduced sediment delivery from comprehensive damming of rivers^21^. Nevertheless, coastal wetlands have a natural capacity to adjust to rising sea level that scientists are just beginning to understand, leading to the suggestion that extreme coastal wetland loss scenarios may be overstated for some natural tidal wetlands^22^. We know even less about sea-level rise influences on created mangrove wetlands, but often we assume long-term persistence when scoping the benefits of climate change mitigation projects.

Tracking soil surface elevation change in created mangrove wetlands over time is critical to assess the longer-term resilience of created mangrove wetlands to sea level, but reliable observations are few. Young mangrove tree roots recolonizing a denuded site in Sydney Harbor, Australia, facilitated soil volume expansion and mineral sedimentation to yield a small soil surface elevation increment (2.9 mm yr^−1^)^23^; however, inter-annual variability in rainfall also exerted influence over surface elevation processes such that the influence of mangrove forest maturity was slightly obscured in this analysis^23, 24^. Mechanistically, increased mangrove plantation density can facilitate surface elevation increases through vertical accretion of sediments, retention of deposited sediments, and root growth^25, 26^. Decomposition rates of leaves and roots can be species-specific and slower in restored sites compared to natural mangrove forests^27^. Thus, it is not surprising that surface elevation change as high as 9.9 mm yr^−1^ has been recorded in at least one mangrove wetland site restored 14–17 years previously^27^; a rate much higher than typically found in older, natural mangrove wetlands^12, 19^. Other restoration activities, such as re-establishing hydrologic connectivity, have resulted in more modest elevation gains (e.g., 2.5 mm yr^−1^ in the Hunter River, Australia)^28^.

Following restoration, early gains in surface elevation may eventually decline, or even reverse, as soils compact and organic matter decomposes over time. Certainly, one would not expect a surface elevation change rate of 9.9 mm yr^−1^ (ref. 27) to be maintained perennially. Indeed, soil organic matter, total nitrogen, and redox potential increased with stand age until approximately 11 years in *Rhizophora mucronata* plantations in the Philippines, and subsequently began to level off with progressive stand maturity^29^. Despite rapid initial soil development, these plantations were estimated to not reach full maturity until approximately 25 years^29^. Documenting surface elevation developmental processes over a range of mangrove wetland ages in relation to sea-level rise has not been attempted anywhere in the world.

Here, we assessed surface elevation change (also referred to recently as vertical land motion, VLM_w_, of the wetland soil surface^20^), vertical accretion of sediments, and sub-surface change (that is, either shallow subsidence from root zone compaction or swelling from root zone expansion) over a five-year period in 18 tidal mangrove wetlands – nine created and nine reference – spanning the geographic range of Tampa Bay, Florida, USA (Supplementary Figure 1). The created mangrove wetlands were originally established as tidal salt marshes, which transitioned naturally to mangrove forests over time; research assessments were established for C storage determination in July 2010^10^. At the time of the first surface elevation change measurements in February 2011, sites ranged in age from 2.4–20.2 yr. Ages at the date of the last measurement reported herein (January 2016) ranged from 7.3–25.1 yr. Sites also differed slightly in elevation (NAVD88) (Supplementary Table 1) and land use history^10^, and were projected to reach functional equivalency to adjacent natural forests in soil bulk density, organic matter, C, and nitrogen in 19–25 years^10^, in close agreement with restoration timelines established for mangrove wetlands in the Philippines^29^.

Tampa Bay represents the largest open-water estuary in the State of Florida, with intertidal wetlands composed of both salt marshes and mangroves. Tides are characterized as lower microtidal (<1 m tidal range), and newly created salt marshes (*Spartina alterniflora*) overtopping sands are naturally colonized by combinations of three mangrove species native to the region: *Rhizophora mangle*, *Avicennia germinans*, and *Laguncularia racemosa*. The order of colonization by these species is somewhat variable, but mixed mangrove communities often develop on these sites^30^.

## Results and Discussion

### Drivers of surface elevation change in created mangrove wetlands

**Figure 1:**
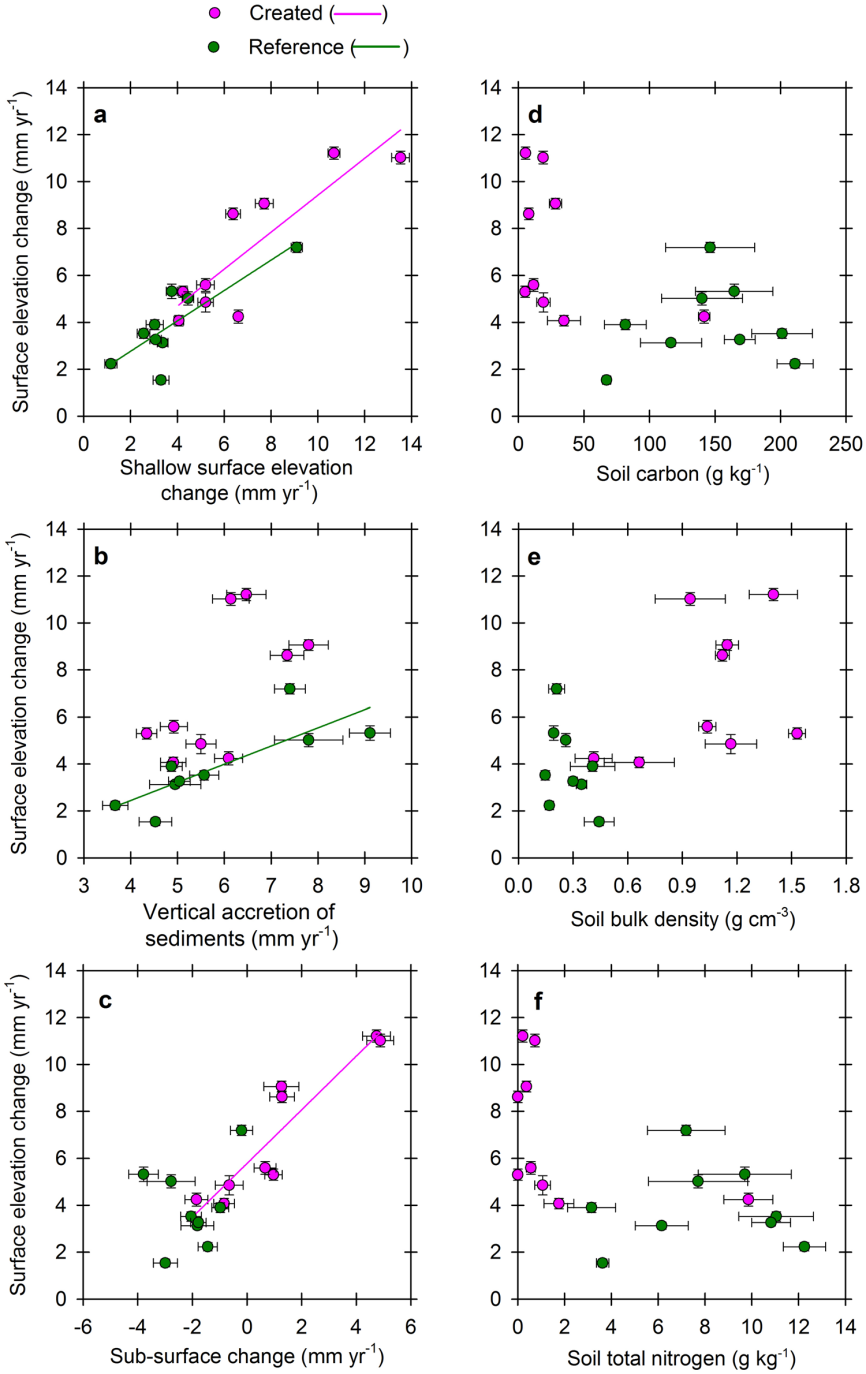
Sedimentation and soil drivers of surface elevation change. Relationship between shallow surface elevation change (**a**), vertical accretion of sediments (**b**), sub-surface change (**c**), soil C content (**d**), soil bulk density (**e**), and soil total nitrogen content (**f**) with surface elevation change from created mangrove wetlands compared with co-located reference mangrove wetlands in Tampa Bay, Florida, USA. Symbols represent means (±SE, bi-directional) of average responses by site, with soil samples (**d**–**f**) representing the average from three plots per site.

**Figure 2:**
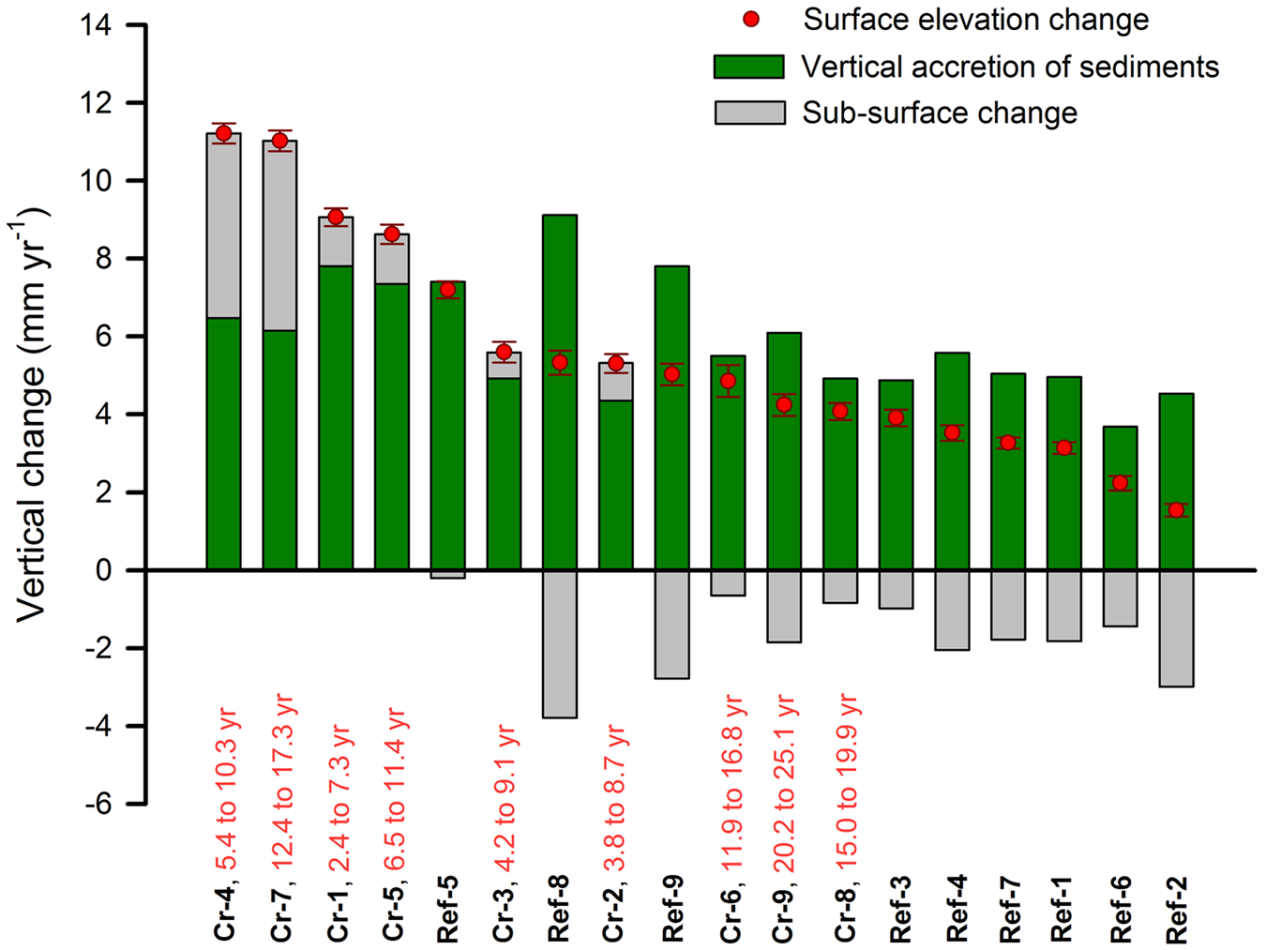
Summary of surface elevation change as related to vertical accretion of sediments versus sub-surface change. Sites are arranged by decreasing rates of surface elevation change. Stacked bars depict the contribution of vertical accretion of sediments and sub-surface change (root volume expansion or compaction) to surface elevation change for nine created mangrove wetlands (Cr) versus nine co-located reference mangrove wetlands (Ref) in Tampa Bay, Florida, USA. Symbols for surface elevation change represent mean (±SE) response per site, or the cumulative contribution of surface accretion combined with sub-surface change. Identical numbers identify created (Cr) and reference (Ref) site pairings.

We suspected that root zone processes would be critical in influencing the surface elevation trajectories on created mangrove wetland sites. We discovered that not only were there no statistical differences between deep surface elevation change measurements (insertion depth of ~8.7 m; *see Materials and Methods*) versus shallow soil surface elevation change measurements (insertion depth of 50 cm) (Fig. 1a; *P* = 0.344, *F* test), but also shallow SET measurements explained 68–75% of the variation in deep SET response. This suggests that the majority of surface elevation change over our measurement period was constrained within the top 50 cm of soil, reiterating a fundamental role for root zone processes in influencing surface elevation patterns in Caribbean mangrove wetlands^31^. Thus, the root zone, which is credited for contributing to C gain in mangrove wetland soils^11^, was responsible for a large percentage of observed surface elevation change – including loss and gain – suggesting that mangrove wetland creation for C sequestration and sea-level rise resilience may be compatible goals into the foreseeable future. A different process, vertical accretion of sediments, influenced surface elevation change strongly on reference sites (*R* = 0.81, *P* = 0.008, *F* test), but not significantly on created sites (*R* = 0.63, *P* > 0.05, *F* test) (Fig. 1b), while the opposite held for sub-surface change (Fig. 1c). Sub-surface change influenced surface elevation change on created sites (*R* = 0.92, *P* < 0.001, *F* test) but not on reference sites (*R* = 0.24, *P* > 0.05, *F* test). Overall, vertical accretion of sediments ranged from 3.7 to 9.1 mm yr^−1^, and sub-surface change ranged from a compaction rate of −3.8 mm yr^−1^ to a root zone expansion rate of 4.9 mm yr^−1^ (Fig. 2; site pairings, actual means, and standard errors are summarized in Supplementary Table 2). Sustained increases in surface elevation as created mangrove wetlands progressed in age over 25 years was driven by a greater capacity for sub-surface expansion than vertical accretion of sediments as sites aged.

Subsurface expansion during the early successional period likely reflects volume expansion from fine and coarse root accumulation, as was also documented in Belizean mangrove wetlands^31^, which overshadowed drivers of shallow subsidence typical of mature forests, such as compaction, root mortality, and decomposition^12^. Development of an organic soil would be a stronger prerequisite for documenting compaction than deposition of pliable mineral sediment in many newly developing mangrove wetland sites. However, tidal wetlands range widely in their reliance on mineral versus biogenic influences to maintain surface elevation gain as they respond to sea-level rise. Soil C, bulk density and total nitrogen concentrations, though sometimes auto-correlated, had no influence on surface elevation change for created or reference mangrove wetlands in Tampa Bay (Fig. 1d–f); however, we expect that this will change over time. Several studies have defined variable relationships between vertical accretion of sediments and surface elevation change from natural mangrove wetlands^31–33^, suggesting mineral sedimentation, root zone, and soil hydration dynamics as co-drivers of surface elevation change. While soil C and total nitrogen concentrations increased^10, 31^ and soil bulk density decreased^10^ as mangroves developed in new areas in the Philippines and Florida, it is noteworthy that newly created mangrove wetlands had a tendency to accrete sediments at very high rates initially^25–27^, followed by later reductions as root-based soils, or peats, were formed, decomposed, and compacted.

**Figure 3:**
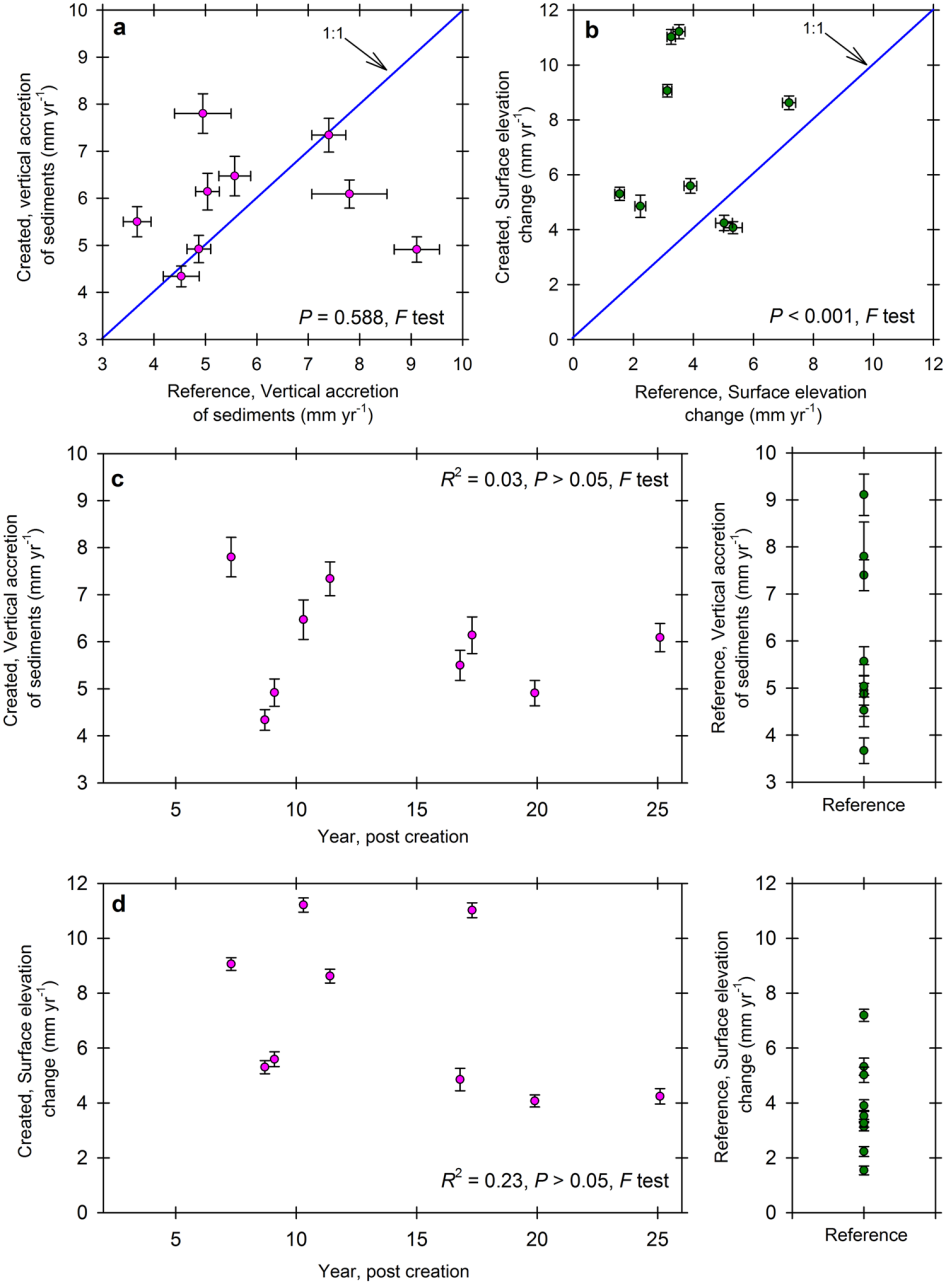
Characteristics of vertical accretion of sediments and surface elevation change. Vertical accretion of sediments (**a**) and surface elevation change (**b**) comparisons for created mangrove wetlands versus their paired reference sites depict a differential reliance on drivers of surface elevation change. Solid blue lines (**a**,**b**) represent a 1:1 relationship for x and y axes. Vertical accretion of sediments (**c**) and surface elevation change (**d**) versus age for created mangrove wetland sites (as in January 2016) relative to naturally established reference mangrove wetland response indicates the trajectory of development toward natural forest condition. Symbols (**a**,**b**) represent means (±SE, bi-directional) of average vertical accretion of sediments or surface elevation change response by site, and means (±SE) of vertical accretion of sediments (**c**) or surface elevation change (**d**) along the age gradient.

Central to surface elevation control on these created mangrove wetlands over time was a shift from the influences of root zone expansion and vertical accretion of sediments for younger mangrove wetlands as new roots occupied soils to an increased influence of shallow subsidence and compaction in comparison to reference sites, and as created mangrove wetlands aged. This is also depicted as higher bulk density values for created mangrove wetlands versus corresponding reference mangrove wetlands of much greater age (Fig. 1e). While vertical accretion of sediments was very high on some created mangrove wetland sites (up to 7.8 mm yr^−1^), rates were not higher than reference sites overall (up to 9.1 mm yr^−1^) (*P* = 0.588, *F* Test), suggesting regional control on vertical accretion of sediments^21^. However, comparative rates of vertical accretion of sediments between created mangrove wetland sites and paired reference mangrove wetlands (within 0.1–2.5 km of each other) were sometimes disparate (Fig. 3a), indicating that in some cases, either local delivery of sediments or the influence of specific root characteristics influenced surface elevation change and may need consideration during placement of future mangrove wetland creation projects globally. With the strong relationship between vertical accretion of sediments and surface elevation change for reference mangrove wetlands (Fig. 1b), and the developing relationship for created mangrove wetlands, positioning projects closer to sources of sediments may affect more rapid early control of surface elevation change. Some mangrove wetlands depend strongly on sedimentation to influence surface elevation change^34, 35^. Also, all three mangrove species produce different root types (i.e., prop roots versus different types of pneumatophores), which can influence deposition, retention, and compaction of sediments^36^.

#### **Mangrove wetland creation, carbon, and current sea-level rise**

**Figure 4:**
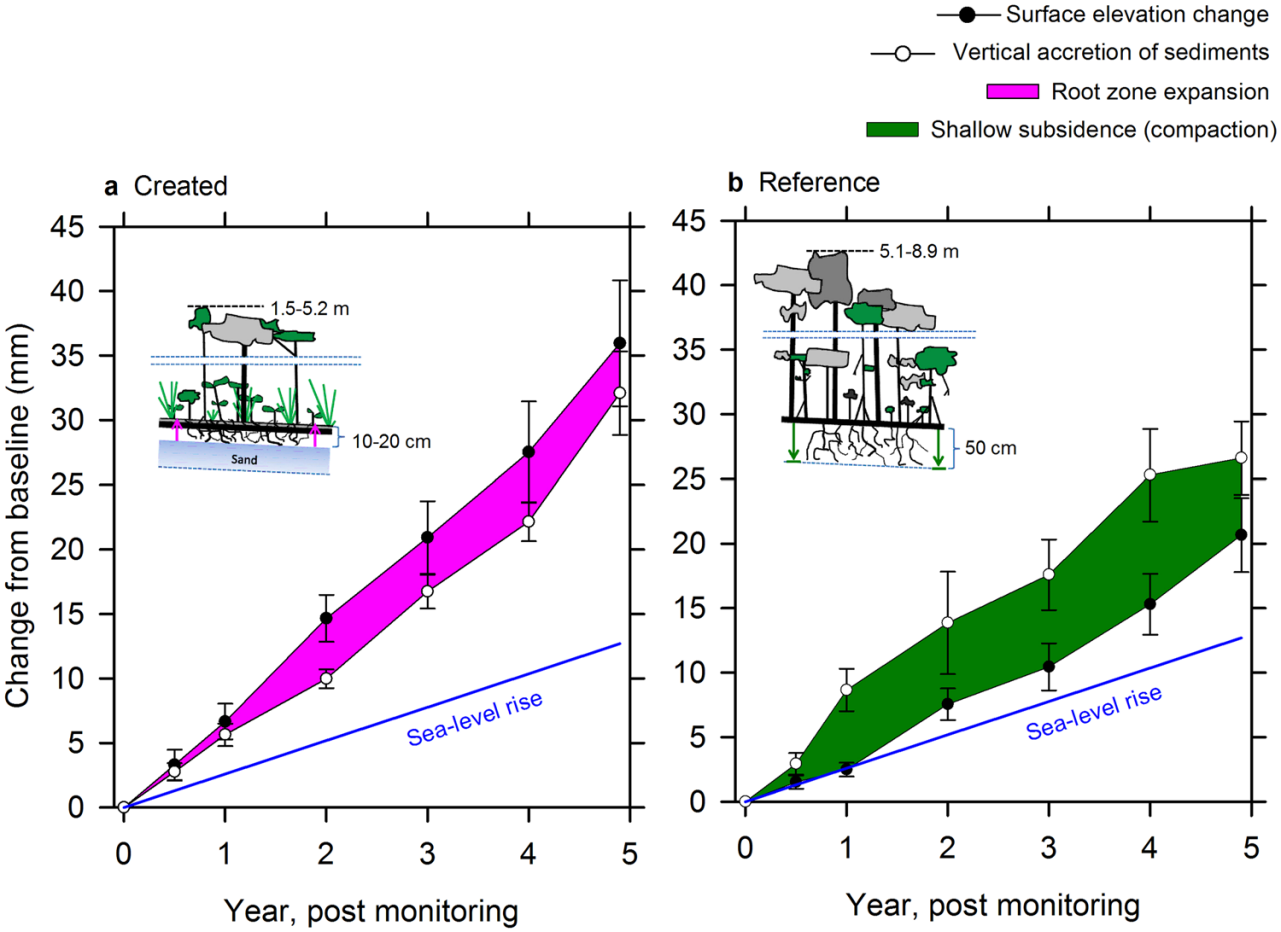
Surface elevation change, vertical accretion of sediments, and root zone expansion versus subsidence. Root zone expansion as the primary driver of surface elevation change on created mangrove wetlands (**a**) is contrasted to shallow subsidence, or root zone compaction, as the primary driver of surface elevation change on reference mangrove wetlands (**b**) in Tampa Bay, Florida, USA. Cartoons depict cross-sections of representative created versus reference mangrove wetlands, including average tree heights and relative influence of the root zone. Symbols represent means (±SE) of the nine created mangrove wetlands and nine reference mangrove wetlands over five years of monitoring.

Over the first 20 years of development, these mangrove wetlands in Tampa Bay accumulated C in the top 10 cm of soil at a rate of 218 g C m^−2 ^ yr^−1^ (ref. 10); therefore, even with little initial capacity for storage of C at depths below 10 cm, C storage paced the 99–226 gC m^−2 ^yr^−1^ reported for tidal wetlands globally^8, 37, 38^. Over the ensuing five years, surface elevation change on these same created mangrove wetland sites averaged 7.2 mm yr^−1^ (±2.9, SE), or roughly 2.7 times current sea-level rise for Tampa Bay. Surface elevation change rates are elevated initially, but settle over time to rates closer to reference mangrove wetlands (Fig. 3d). The higher rate of surface elevation change was influenced greatly by the expansion of the root zone on created mangrove wetland sites (Fig. 4a), which pushes up on the soil surface from the amassing of subaerial root material as vegetation colonizes and develops on these new sites^18^. The sheer density of aerial roots and herbaceous shoot structures, which expand in diameter just at the soil surface and position fine roots to retain deposited sediments, also influence surface elevation gain^36, 39^. For example, root volume expansion was responsible for 1.2 to 10.8 mm yr^−1^ of surface elevation change in a low-sediment Caribbean carbonate system^31^. The capacity for positive surface elevation change was much stronger for created mangrove wetlands than their corresponding reference forests; although this was not predictable through polynomial regression (R^2^ = 0.23, *P* > 0.05, *F* test) (Fig. 3b). Persistent root zone expansion was replaced over two decades by a greater reliance on mineral deposition to offset root zone compaction, or subsidence, as created mangrove wetlands developed further with age. Surface elevation change for reference mangrove wetlands in Tampa Bay averaged 3.9 mm yr^−1^ (±1.7, SE), which was much less than observed for the created mangrove wetlands (*P* < 0.001, *F* test), and only 1.5 times higher than current sea-level rise (Fig. 4b). Thus, while vertical accretion of mineral sediments remained similar on created versus reference sites, root zone expansion was replaced by root zone compaction over time. In keeping with this trend, bulk density also decreased with created mangrove wetland age to approximate natural forests in 25 years^10^, reflecting the greater compaction capacity over time as greater organic fractions contributed to soil structure.

Experimental manipulations of mangrove tree plantings in Sri Lanka suggest much wider application of our results from Tampa Bay. There, *Rhizophora mucronata* plantation densities were manipulated in order to relate surface elevation dynamics, vertical accretion of sediments, and vegetation influences^25^. Vertical accretion of sediments related positively to seedling density, and increased for approximately two years, leveling off as surface elevation change (that is, roughly equivalent to shallow surface elevation change in our study) reached a threshold elevation between vertical accretion of sediments and root zone expansion. Soils were not yet compactable and influenced strongly by root in-growth, somewhat like our younger created mangrove wetlands in Tampa Bay. Furthermore, the mix of marsh grass (*Spartina alterniflora*), forbs (for example, *Batis maritima*), and mangrove seedlings of various species (*R*. *mangle*, *A*. *germinans*, and *L*. *racemosa*) and colonization trajectories, growth rates, and sizes on created mangrove wetlands in Tampa Bay potentially contributed to overyielding of belowground volume increment (and positive surface elevation change), as has also been demonstrated in young mangrove wetland restoration sites in Kenya^40^. Root growth can be prolific as mangrove seedlings grow in close association, influencing surface elevation change through facilitated accretion and root volume expansion at higher densities (up to ~7 seedlings m^−2^)^26^. The degree to which such influences level-off is unknown, but based on the trajectory of surface elevation change development for created mangroves in Tampa Bay versus reference mangrove wetland surface elevation change response, that shift might occur around 25 years (Fig. 3c,d), corresponding nicely to well-described measures of biomass increases in mangrove forestry^41, 42^.

Curiously, some tidal wetlands can accumulate C in soils while simultaneously experiencing surface elevation deficits. One example includes tidal freshwater forests, which from several locations along the south Atlantic coast of the U.S., accumulate C in soils at rates of 49–82 g C m^−2 ^yr^−1^, while simultaneously remaining quite susceptible to sea-level rise^43^. Based upon an empirical model presented in Lovelock *et al*.^21^, which considers tidal range, intertidal position, and local surface elevation deficit, many tidal wetlands might yet be around for several additional centuries even with surface elevation deficits of 2–4 mm yr^−1^; tidal range, intertidal position, and the rate of local sea-level rise are key. The tidal range for Tampa Bay is only 0.67 m^44^, which might limit migration capacity for mangrove wetlands by restricting the intertidal area and forcing a greater reliance on *in-situ* surface elevation gains. How might a reduction in surface elevation change as created mangrove wetlands develop over time influence long-term susceptibility to sea-level rise in Tampa Bay? Simplistically, surface elevation change is higher than current sea-level rise for Tampa Bay (Fig. 4); however, this comparison excludes IPCC projections of increased rates of sea-level rise into the future^45^ and assumes a static elevation devoid of known biogenic feedbacks among vegetation productivity and vertical accretion of sediments as influenced by the elevation of the wetland relative to current sea level^46^. Such direct comparisons using linear relationships can overestimate wetland change^22^, justifying the use of modeling to answer this question fully.

#### **Modeling: future perspectives assuiming accelerated sea-level rise**

**Figure 5:**
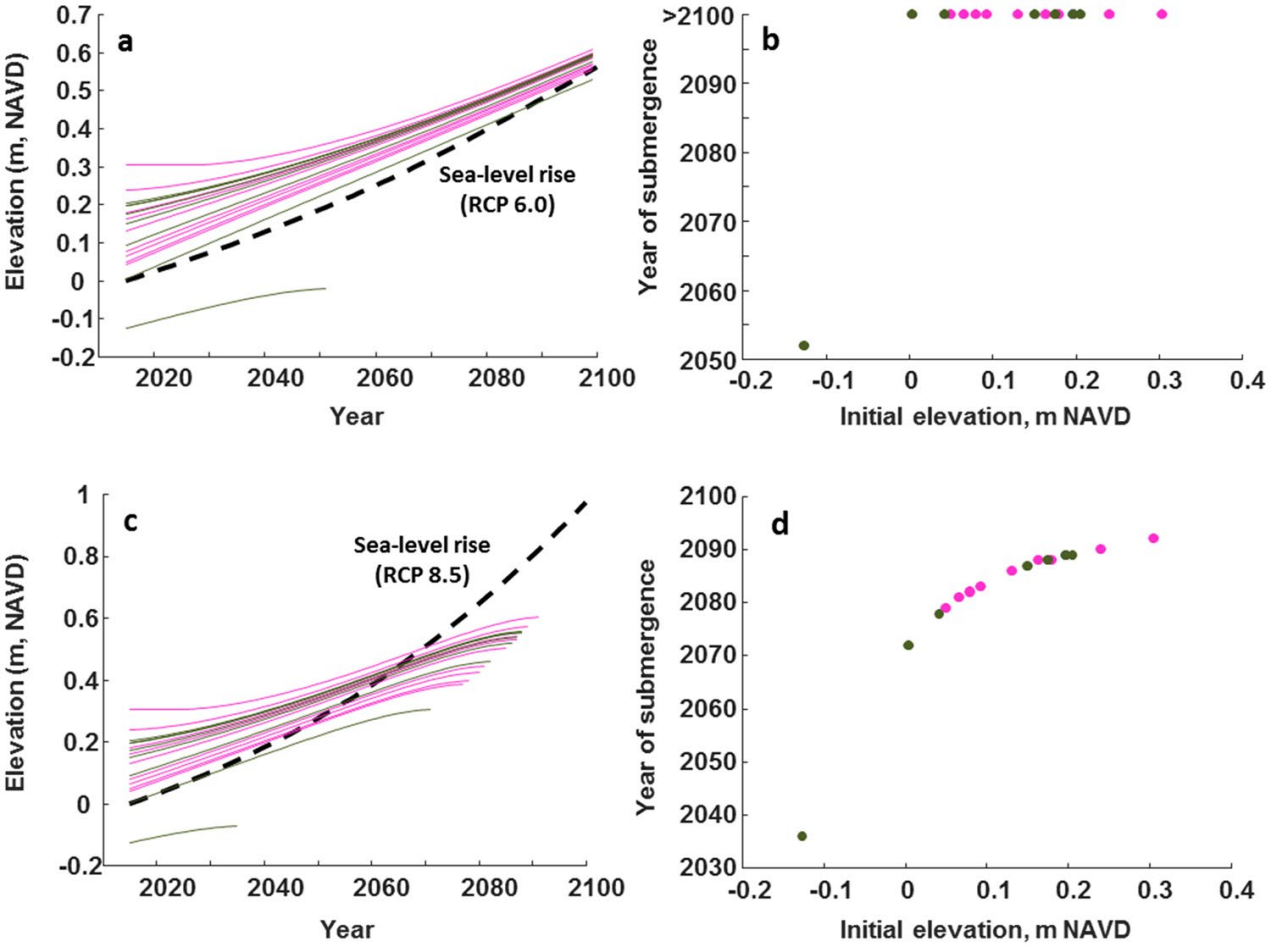
Modelled year of submergence versus starting elevation (m NAVD88) for two accelerated sea-level rise scenarios. Projected year of submergence versus site elevation as a continuous (**a**) and discrete (**b**) function for medium sea-level rise projections (dashed line, IPCC RCP 6.0; 0.55 m by 2100), and projected year of submergence versus site elevation as a continuous (**c**) and discrete (**d**) function for fast sea-level rise projections (dashed line, IPCC RCP 8.5; 0.74 m by 2100) for nine created mangrove wetlands (pink lines and symbols) versus nine co-located reference mangrove wetlands (green lines and symbols) in Tampa Bay, Florida, USA.

We considered two sea-level rise acceleration scenarios: a medium acceleration (0.55 m by 2100; IPCC RCP 6.0) and a high acceleration (0.74 m by 2100; IPCC RCP 8.5). All 18 mangrove wetland sites – created and reference – were considered during modeling scenarios; increases in site elevation (NAVD88) relative to sea-level rise occurs as either a surplus or deficit over time, such that for the latter, drowning might occur related to the capacity for maximum surface elevation change recorded from these sites. For the medium sea-level rise scenario (RCP 6.0), only a single site is projected to be lost by 2052 (Fig. 5a), demonstrating the importance of vertical position within the intertidal frame at the beginning of simulations^21^. This site had the lowest starting elevation of −0.1265 m NAVD88 (Fig. 5b); despite surface elevation change adjustment of 3.9 mm yr^−1^, this acceleration in sea-level rise pressed this one site to submerge fairly quickly. Sea-level rise rates of 5.2 mm yr^−1^ documented through part of the Holocene (specifically 10,600–7,700 yr BP) for the western Atlantic^47^ also inhibited mangrove colonization over that period, until sea-level rise rates slowed to create accommodation space for colonization^31, 48^. In addition, elevations across sites are converging through time, but a near constant slope after about 2070 indicates that all sites are near the maximum rate of surface elevation change (Fig. 5a) based on the empirical relationship developed here-in (Supplementary Figure 2), with no differentiation between submergence dates for created versus reference mangrove wetlands (Fig. 5b). Under this medium scenario, no created mangrove wetland site was projected to submerge before 2100.

For the fast sea-level rise scenario (RCP 8.5), all sites are projected to submerge by 2100 (Fig. 5c), ranging in date of submergence from 2079–2092 for created mangrove wetlands to 2036–2089 for reference mangrove wetlands (Fig. 5d). Thus for RCP 8.5, the initial elevation (NAVD88) of the mangrove wetland site did not affect whether the mangrove wetland submerged before 2100, but rather, initial site elevation influenced the year in which submergence would occur. Together, these results suggest that created and natural mangrove wetlands will eventually build at similar rates, and that wetlands created at relatively high elevations may survive sea level rise longer than natural wetlands among a range of SLR scenarios.

Considering the date of mangrove wetland creation and the rate of soil C storage documented on these sites (218 g C m^−2 ^yr^−1^)^10^, total soil C storage in these created mangrove wetland sites in Tampa Bay since establishment in 1990–2008 would range from 201 to 240 Mg C ha^−1^ through 2100 with current sea-level rise and RCP 6.0 (medium) sea-level rise acceleration scenarios. Similarly, assuming that all C is lost when created mangrove wetlands submerge, total soil C storage would be reduced to 155 to 214 Mg C ha^−1^ among sites through 2100 under an RCP 8.5 (fast) sea-level rise acceleration scenario. In addition, for current and RCP 6.0 scenarios, soil C sequestration would continue beyond 2100; however, the relationship between mangrove wetland site submergence and soil C loss after submergence under the RCP 8.5 scenario is not fully understood. Landward migration of created and natural mangrove wetlands would generate additional opportunities for soil C storage as long as sea-level rise acceleration is not too fast and migration corridors are available^49–51^.

## Materials and Methods

### Background

Study sites in Tampa Bay were originally established to understand how long it takes created tidal wetlands, transitioning naturally from marsh to mangrove wetlands, to become functionally equivalent to adjacent natural reference mangrove wetlands. Original structural metrics for rating functional equivalency included marsh aboveground biomass, marsh plant stem density, juvenile mangrove tree height, juvenile mangrove tree density, adult tree diameter, and adult tree density, while soil metrics included bulk density, sand content, moisture, organic matter, C, and nitrogen^10^ (see “Soil sampling”, Supplementary Information). We are adding measurements of vertical accretion of sediments and surface elevation change to this assessment, and broadening the scope and inference through modeling.

### Study sites and experimental design

Projects aimed at restoring mangrove function globally are termed many things^52^, but all attempt to restore the ecological functions that mangrove wetlands once provided to individual coastlines. All created sites in Tampa Bay represent newly created intertidal areas that were graded to tidal elevation using a mix of upland soils atop underlying sand, planted with marsh grass (*Spartina alterniflora*)^53^, and seeded naturally with mangroves^13^. Initially, the newly graded soils of the youngest sites had very little organic matter (<2%) and a high percentage of sand (>60%)^10^, relying on root development from vegetation to increase organic matter content over time. Because of an abundant propagule supply from all three primary mangrove tree species in the region (*Rhizophora mangle*, *Laguncularia racemosa*, *Avicennia germinans*), mangroves began to colonize sites within three years and eventually shaded out marsh grasses by 11–12 years^10^.

We used a space-for-time substitution approach^54^ by identifying creation projects that were previously completed using reasonably similar approaches but that were also of different ages. Sites were created between 1990 and 2008, and selected to be adjacent to nearby natural mangrove wetlands that were seemingly unstressed to use as reference sites (Supplementary Figure 1, Supplementary Table 1). Reference sites likely vary considerably in age. Along with the original space-for-time approach, we also re-measured plots annually over 5 years to establish the equivalent of a 25 year record of vertical accretion of sediments and surface elevation change.

### Surface elevation change and sedimentation processes

Surface elevation change and vertical accretion of sediments on each mangrove wetland site were measured using the Surface Elevation Table – Marker Horizon (SET-MH) approach^55^. The SET was modified through subsequent designs^56^, and makes use of rods driven to National Geodetic Survey (NGS) refusal standards for Class B rods^57^, for straight-forward comparisons with regional tide gauges (rod SET, hereafter SET, see conceptual diagram in ref. 20). Thus, tide gauge records of relative sea-level rise (RSLR) combined with SET measurements of surface elevation change, allow for calculations of wetland-specific rates of relative sea-level rise (RSLR_wet_), using the following formula^20^:

1 $${{\rm{RSLR}}}_{{\rm{wet}}}=\mathrm{RSLR}-\mathrm{surface}\,{\rm{elevation}}\,{\rm{change}}$$

It is important to make the distinction for wetland scientists that tide gauge readings include surface elevation change and vertical land motion from glacial isostatic adjustment and tectonics^20^ and represent the more common measure of RSLR used globally. Both RSLR_wet_ and RSLR are relative, but to different things. Because we are ultimately interested in RSLR_wet_ for created mangrove wetland sites in Tampa Bay, we use the SET approach to isolate and track surface elevation change (see “SET-MH installation and procedure”, Supplementary Information, for additional SET-MH details).

### Determination of current sea-level rise

Current, relative sea-level rise (RSLR) for Tampa Bay was determined as a linear increase in monthly mean sea level (meters) with the average seasonal cycle removed. Data were from National Oceanic and Atmospheric Administration (NOAA) Gauge #8726520 (St. Petersburg, Florida), and trends have been archived since January of 1947 (http://tidesandcurrents.noaa.gov). The mean 69-year RSLR trend for Tampa Bay is 2.6 mm yr^−1^ (±0.25 mm yr^−1^, 95% Confidence Interval, downloaded January 2016). This gauge is surveyed against terrestrial benchmarks inserted to depths of 14.6 m (6520 H 1993), 10.9 m (6520 J 1997), and 13.1 m (6520 K 1998), along with a number of shallow benchmarks to confirm stability. Thus, it is assumed that vertical land motion from glacial isostatic adjustment and tectonics at the SETs and tide gauge survey datum cancel, with the difference in measurements of surface elevation change and RSLR equating to RSLR_wet_^20^ (see “Elevation Surveys and Soil Sampling”, Supplementary Information, for details on RTK elevation and soil surveys).

### Empirical modeling

We considered two sea-level rise acceleration scenarios: a medium acceleration (0.55 m by 2100; IPCC RCP 6.0) and a high acceleration (0.74 m by 2100; IPCC RCP 8.5), both superimposed on a local subsidence rate of 0.9 mm yr^−1^ inferred from tide gauges. A simple, empirical relationship depicting five-year vertical accretion of sediments and surface elevation change response from our reference forests versus absolute elevation (NAVD88) was developed to support the model, and indicates a feedback whereby the lower elevation site (−0.1265 m, NAVD88) and the higher elevation sites (0.2049–0.3039 m, NAVD88) have a reduced capacity for surface elevation adjustment and vertical accretion of sediments (Supplementary Figure 2), while surface elevation change and vertical accretion of sediments are maximized at moderate elevations. This curvilinear relationship for mangrove wetlands fits similar to productivity-inundation and sedimentation-inundation algorithms developed for marshes^39, 46, 58, 59^; however, threshold elevations for mangrove wetlands are expected to be characteristically higher^60, 61^, though to date, have been undefined specifically. From this, a simple model was developed to predict the response of mangrove wetland elevation to accelerated sea-level rise over the 21^st^ century (see “Empirical Model Development”, Supplementary Information, for additional model details).

### Statistical analyses

From each of the two SETs per site, four directions were measured as an average of nine pin measurements incorporating soil micro-topographic variability. Not all pins were available for each measurement period, and can be obscured by oyster shells, crab holes, mangrove prop roots, and other objects (see raw data, link provided under “Additional information”). Thus, each SET provided four surface elevation change measurements, from up to nine observations of each measurement. The four directions were initially treated as sub-samples, and thus, the SETs were treated as the experimental unit; directions were nested within each experimental unit to avoid pseudoreplication^62, 63^ and to adjust for sub-sampling. However, we found that for all analyses the variance component for “direction(SET)” was significant (*P* < 0.001, *F* test), indicating that the all eight directions could be considered independent samples, allowing us to drop the nesting term and treat directions as samples (n = 8 per site). Shallow-SET data were treated similarly, except that only four directions were available as sample replicates for shallow-SETs. Three independent measures of vertical accretion of sediments were made from feldspar MH plots per SET (n = 9 per site). Sub-surface change is a derived variable, calculated as the difference between surface elevation change (using the SET) and vertical accretion of sediments (using the MH).

Standard regression procedures were used to test the null hypothesis that SET, shallow-SET, vertical accretion of sediments, and sub-surface change trends did not differ significantly from a slope of 0 over time. Slopes were further analyzed using ANOVA techniques for differences among sites, and for differences between created and reference mangrove wetlands, using a general linear model (Type 1 error valid for regression models with categorical variables)^34^. Correlation analyses were used to determine inter-relatedness among measures of surface elevation change, vertical accretion of sediments, and soil properties. All data were analyzed using SAS (Version 9.4, SAS Institute, Cary, North Carolina, USA), and the residuals of each model were normal with homogeneous variances without transformation.

## Electronic supplementary material

Supplementary Information: Created mangrove wetlands store belowground carbon and surface elevation change enables them to adjust to sea-level rise
