# Supplementary material for: Created mangrove wetlands store belowground carbon and surface elevation change enables them to adjust to sea-level rise
Source: Sci Rep. 2017 Apr 21;7:1030. doi: 10.1038/s41598-017-01224-2 (PMC5430729; doi:10.1038/s41598-017-01224-2)
Supplement: Supplementary file 1 — Supplementary Information: Created mangrove wetlands store belowground carbon and surface elevation change enables them to adjust to sea-level rise [file 41598_2017_1224_MOESM1_ESM.pdf]

SUPPLEMENTARY INFORMATION

Created mangrove wetlands store belowground carbon and surface elevation change enables them to adjust to sea-level rise

Ken W. Krauss[1*], Nicole Cormier[1], Michael J. Osland[1], Matthew L. Kirwan[2], Camille L. Stagg[1], Janet A. Nestlerode[3], Marc J. Russell[3], Andrew S. From[1], Amanda C. Spivak[4], Darrin D. Dantin[3], James E. Harvey[3], and Alejandro E. Almario[3]

[1]U.S. Geological Survey, Wetland and Aquatic Research Center, 700 Cajundome Blvd., Lafayette, Louisiana 70506, USA. [2]Virginia Institute of Marine Science, College of William and Mary, Gloucester Point, Virginia 23062, USA. [3]U.S. Environmental Protection Agency, Gulf Ecology Division, 1 Sabine Island Drive, Gulf Breeze, Florida 32561, USA. [4]Woods Hole Oceanographic Institution, Marine Chemistry and Geochemistry, 266 Woods Hole Rd., Woods Hole, Massachusetts 02543, USA.*e-mail: kraussk@usgs.gov

SET-MH installation and procedure. SETs were installed in January 2011. All SET rods reached NGS refusal standards after being driven to at least 8.2-8.7 m into the soil. Rods were 1.43-cm-diameter stainless steel, joined in 1-m sections and designed to screw into each other for adding length to reach the point of refusal. Rods were driven mechanically using an electric demolition hammer powered by a portable generator. In most cases, underlying carbonate rock was hit and rods were halted abruptly. Rods were encased in a 15.2-cm-diameter PVC pipe dug approximately 70 cm into the soil, attached with an SET receiver, and cemented into place. The SET arm itself is portable, and fits onto the permanent SET receiver in the exact same location each time that measurements are made.

Sites were accessed rarely, and SET measurements were always made using portable, long metal planks with bases outside of SET plots and measurement influence. Each SET arm has nine fiberglass pins, which after being attached to the receiver and leveled horizontally, are lowered to the soil surface in the same place every time measurements are made. Confidence intervals associated with repetitive SET measurements were previously reported as ± 1.3 mm[56], and the same person (N.C.) took all measurements throughout. For each SET, four SET arm directions were measured each time for a total of 36 observations. In addition, we installed two replicate SETs on each site, which generated 72 measurements of surface elevation change, but with a mix of interdependence (see Statistical Analysis). Measurements were made initially and annually for five years spanning 2011 to 2016, across all 18 sites.

In addition to paired SETs that measured surface elevation change associated with an ~8.7 m depth, we also installed a single shallow-SET on each site. The shallow-SET represents another design modification to the original SET[56]. Ours were fitted with four, 7.6-cm-diamater legs for stability, all inserted to a soil depth of 50 cm, or the estimated root zone depth as assessed from cores taken on the reference sites. The root zone extended to no more than 10 cm on the created mangrove wetlands over the first 20 years[10]. The same SET arm attaches to the shallow-SET, and the same procedures are followed. The shallow-SET allows us to isolate the contributions of the root zone from the full 8.2-8.7 m surface elevation change profile.

Three replicate feldspar marker horizons (MH) were laid within three meters of each of the three SETs (two paired SETs, one shallow-SET) to estimate vertical accretion of sediments over

the same increments as SET measurements[64]. MH layers were marked and sampled using a miniature Russian peat corer[65], modified to a diameter of 2.5 cm and a 17.8 cm core length. The amount of sediment deposited above the MH was measured using a hand ruler upon extraction of each core. When cores are extracted, only cores having a clearly defined MH are measured, which can create a slight positive bias initially until MH layers are buried because eroded layers cannot be measured[66].

Sub-surface change was measured as the difference between surface elevation change and vertical accretion of sediments[66]. When surface elevation change < vertical accretion of sediments (*negative* values), sub-surface change is from shallow subsidence or compaction; when surface elevation change > vertical accretion of sediments (*positive* values), sub-surface change is from root zone expansion. Root zone expansion can occur when root growth is so vigorous that soil volume is also affected[31]. Between the space-for-time substitution design of our experiment (i.e., oldest site at time of SET installation, 20.2 yr) and the five-year measurement period, we establish a 25 year record of surface elevation change observations. Through modeling, we extrapolate SET responses to 2100; therefore, we use 25 years of SET observations to project 84 years into the future.

Empirical model development. Empirical model projections were conducted in Matlab (The MathWorks, Inc., Massachusetts, USA), and were simple projections of field measurements. Modeled rates of elevation change (dz/dt) depend on elevation relative to sea level (z), according to the empirical, quadratic function:

$$dz/dt = az^2 + bz + c \qquad [2]$$

where a = -112.0401, b = 3.6304, and c = 6.2619. This relationship is based on the long-term surface elevation change rates measured at eight of the reference mangrove wetlands in Tampa Bay (Supplementary Figure 2b); we could not establish NAVD88 elevations for the ninth reference site at Emerson Point. Following common salt marsh models[39,58,61], this approach assumes that rates of elevation gain are maximized at an optimum elevation relative to sea

level, and decrease towards the upper and lower elevation limits of mangrove growth. The maximum surface elevation change predicted in the model is 6.3 mm yr^{-1}, which corresponds closely to the elevation change rate measured at our Bullfrog Creek reference mangrove site (7.19 mm yr^{-1}, Supplementary Table 2).

The response of surface elevation change to sea level rise was modeled using two IPCC eustatic sea-level rise scenarios adjusted for local subsidence. In the first model simulation, sea level rises according to the median sea-level rise in the RCP 6.0 emissions scenario (0.55 m in 2100 relative to 1996), hereafter referred to as "medium" scenario. In the second model simulation, sea level rises according to the median RCP 8.5 scenario (0.74 m in 2100 relative to 1996), hereafter referred to as "high" scenario. These IPCC sea level scenarios were adjusted so that model simulations began with a sea level of 0 m in 2015. In both simulations, a constant rate of subsidence (0.9 mm yr^{-1}) was added to eustatic projections, which reflects the difference between 20th century eustatic rates (1.7 mm yr^{-1}) and the historical relative sea-level rise rate measured at the St. Petersburg, Florida, tide gauge through January of 2016 (2.6 mm yr^{-1}) (https://tidesandcurrents.noaa.gov, accessed January 2016). Total sea-level rise during the 85 year model simulation was 0.56 m and 0.97 m in the medium and high scenarios, respectively. Maximum rates of sea-level rise in 2100 were 8.5 mm yr^{-1} (medium) and 18.3 mm yr^{-1} (high).

Model simulations begin in 2015 with elevations measured in 2014-2015 at each study site. Rates of elevation change for each site are calculated with an annual time step, and simulations stop in 2100 or whenever the elevation of the mangrove wetland relative to sea level falls below the minimum required for persistent mangrove survival, hereafter referred to as the "year of submergence" (see also ref. 21). Furthermore, NAVD88 elevations from our sites in Tampa Bay come close to spanning the full range of acceptable elevations for mangrove wetland development in south Florida, representing an elevation gradient from near terrestrial (0.3030 m, NAVD88) to just at the transition between mangrove wetland and marsh (-0.1265 m, NAVD88). This lower elevation limit is based on field based surveys of the marsh-mangrove ecotone along the SW Florida coast which indicate that the minimum elevation for mangrove wetland occurrence is approximately -0.21 m NAVD88, beyond which marshes persist (G. Anderson, U.S. Geological Survey, unpublished data).

Elevation surveys and soil sampling. The soil surface of all sites was surveyed during two separate field expeditions using either a Trimble R8 (2014) or Trimble R10 (2015) Global Navigation Satellite System (GNSS) (Trimble Navigation Limited, Sunnyvale, California, USA). Each survey was conducted using the receiver (R8 or R10) as a static survey base station for a minimum of two hours, while using a Trimble DiNi Digital Level to perform the horizontal survey under the forest canopy and tying into the position of the Trimble receiver to calculate elevation. Each survey file was downloaded and processed through OPUS: Online Positioning User Service (https://www.ngs.noaa.gov/OPUS/) which uses data available from a nearby Continuously Operating Reference Station (CORS) to calculate latitude, longitude, and elevation. The elevation is given in relationship to the North American Vertical Datum of 1988 (NAVD88), in meters, along with the geoid model used, in our case GEOID12A.

Soils were sampled at the beginning of this study[10]. Each site contained a total of three 100-m^2 plots to support measurements, and four soil cores were collected within each plot to a depth of 30 cm. 4.7-cm-diameter cores were collected with a stainless steel split-cylinder corer, sectioned into 0-10 and 10-30-cm increments, and analyzed for bulk density, C, and nitrogen. Of the four cores collected on each plot, one was used for depth-specific bulk density determination, and the other three were composited by depth increment, resulting in a sample size of three per site. Soils were kept on ice or refrigerated until analysis. Bulk density was determined on samples dried to a constant weight at 105 °C using a dry weight to volume ratio[67]. For determination of total C and nitrogen, soils were sieved, dried, and homogenized with an analytical mill, and measured using dry combustion techniques[68-69]. Soil total C and total nitrogen analyses were conducted by the U.S. Environmental Protection Agency (Mid-Continent Ecology Division).

References

64. Cahoon, D. R. & Turner, R. E. Accretion and canal impacts in a rapidly subsiding wetland. II. Feldspar marker horizon technique. *Estuaries* **12**, 260–268 (1989).

65. Jowsey, P. C. An improved peat sampler. *New Phytol.* **65**, 245–248 (1966).

66. Cahoon, D. R. & Lynch, J. C. Vertical accretion and shallow subsidence in a mangrove forest of southwestern Florida, USA. *Mangroves Salt Marshes* **1**, 173–186 (1997).
67. Blake, G. R. & Hartge, K. H. in *Methods of Soil Analysis, Part 1. Physical and Mineralogical Methods* (ed Klute, E.) 363–375 (American Society of Agronomy and Soil Science Society of America, 1986).
68. Tiessen, H. & Moir, J. O. in *Soil Sampling and Methods of Analysis* (ed Carter, M.R.) 187–199 (Canadian Society of Soil Science, 1993).
69. McGill, W. B. & Figueiredo, C. T. in *Soil Sampling and Methods of Analysis* (ed Carter, M.R.) 201–211 (Canadian Society of Soil Science, 1993).

Supplementary Table 1 | Characteristics of created and natural reference tidal mangrove sites in the Tampa Bay region of Florida, USA.

Code[a]	Site name	Type	Month created	Year created	Age (yr), Feb 2011	Age (yr), Jan 2016	Tree height (m ± SE)	Elevation (m ± SE, NAVD88)	Latitude		Longitude	
Cr-1	Bishop Harbor	Created	September	2008	2.4	7.3	1.50 ± 0.08	0.0496 ± 0.009	27.60208	N	-82.55324	W
Cr-2	Newman Branch	Created	May	2007	3.8	8.7	1.60 ± 0.11	0.3039 ± 0.005	27.78246	N	-82.40245	W
Cr-3	Robinson Preserve	Created	December	2006	4.2	9.1	3.86 ± 2.04	0.1631 ± 0.003	27.52026	N	-82.66906	W
Cr-4	Braided Tidal Creek	Created	September	2005	5.4	10.3	1.68 ± 0.15	0.0916 ± 0.004	27.69200	N	-82.50674	W
Cr-5	Schultz Preserve	Created	August	2004	6.5	11.4	2.16 ± 0.12	0.2389 ± 0.008	27.81314	N	-82.39665	W
Cr-6	Frog Pond	Created	April	1999	11.9	16.8	2.87 ± 0.19	0.1303 ± 0.002	27.69295	N	-82.51068	W
Cr-7	Emerson Point	Created	October	1998	12.4	17.3	2.61 ± 0.15	0.0658 ± 0.017	27.53425	N	-82.62665	W
Cr-8	Stormwater	Created	February	1996	15.0	19.9	3.26 ± 0.38	0.0787 ± 0.011	27.68454	N	-82.49859	W
Cr-9	E.G. Simmons	Created	December	1990	20.2	25.1	5.19 ± 0.22	0.1798 ± 0.007	27.74466	N	-82.47232	W
Ref-1	Bishop Harbor	Reference	--	--	--	--	5.14 ± 0.45	0.1968 ± 0.014	27.60244	N	-82.56005	W
Ref-2	Apollo Beach	Reference	--	--	--	--	4.46 ± 0.22	0.1973 ± 0.004	27.78231	N	-82.41685	W
Ref-3	Robinson Preserve	Reference	--	--	--	--	4.27 ± 0.25	-0.1265 ± 0.007	27.52014	N	-82.66956	W
Ref-4	South Cockroach Bay	Reference	--	--	--	--	6.32 ± 0.50	0.1494 ± 0.004	27.66975	N	-82.51480	W
Ref-5	Bullfrog Creek	Reference	--	--	--	--	8.88 ± 0.82	0.0036 ± 0.003	27.83467	N	-82.38978	W
Ref-6	Little Cockroach Bay	Reference	--	--	--	--	7.85 ± 0.72	0.2049 ± 0.008	27.69520	N	-82.51209	W
Ref-7	Emerson Point	Reference	--	--	--	--	4.68 ± 0.41	--	27.53445	N	-82.62664	W
Ref-8	East Cockroach Bay	Reference	--	--	--	--	5.78 ± 0.37	0.0414 ± 0.027	27.67662	N	-82.50331	W
Ref-9	Wolf Branch	Reference	--	--	--	--	6.55 ± 0.55	0.1744 ± 0.004	27.73984	N	-82.46202	W

SE = Standard error of 3-6 locations for each plot separated by two separate measurement attempts in time (February 2014 and 2015).
[a] Identical numbers identify created (Cr) and reference (Ref) site pairings.

Supplementary Table 2 | Component measurements of surface elevation response of created and reference tidal mangrove sites over five years in the Tampa Bay region of Florida, USA.

							Deep rSET[b]				Shallow SET[d]	
Code[a]	Site name	Type	Age (yr), Feb 2011	Age (yr), Jan 2016	Vertical accretion of sediments mm yr^{-1}	*P* > F	Surface elevation change, mm yr^{-1}	*P* > F	[c]Sub-surface change, mm yr^{-1}	*P* > F	Shallow surface elevation change, mm yr^{-1}	*P* > F
Cr-1	Bishop Harbor	Created	2.4	7.3	7.80 ± 0.42	***	9.06 ± 0.23	***	1.26 ± 0.64	ns	7.71 ± 0.38	***
Cr-2	Newman Branch	Created	3.8	8.7	4.34 ± 0.22	***	5.30 ± 0.24	***	0.97 ± 0.32	**	4.24 ± 0.19	***
Cr-3	Robinson Preserve	Created	4.2	9.1	4.92 ± 0.29	***	5.59 ± 0.27	***	0.66 ± 0.40	ns	5.20 ± 0.38	***
Cr-4	Braided Tidal Creek	Created	5.4	10.3	6.47 ± 0.42	***	11.21 ± 0.26	***	4.74 ± 0.51	***	10.69 ± 0.25	***
Cr-5	Schultz Preserve	Created	6.5	11.4	7.34 ± 0.36	***	8.62 ± 0.25	***	1.28 ± 0.45	**	6.38 ± 0.32	***
Cr-6	Frog Pond	Created	11.9	16.8	5.50 ± 0.32	***	4.85 ± 0.41	***	-0.65 ± 0.51	ns	5.21 ± 0.33	***
Cr-7	Emerson Point	Created	12.4	17.3	6.14 ± 0.39	***	11.02 ± 0.27	***	4.88 ± 0.49	***	13.53 ± 0.38	***
Cr-8	Stormwater	Created	15.0	19.9	4.91 ± 0.27	***	4.07 ± 0.22	***	-0.84 ± 0.38	*	4.06 ± 0.21	***
Cr-9	E.G. Simmons	Created	20.2	25.1	6.09 ± 0.30	***	4.24 ± 0.28	***	-1.85 ± 0.42	***	6.60 ± 0.15	***
Ref-1	Bishop Harbor	Reference	--	--	4.95 ± 0.55	***	3.13 ± 0.15	***	-1.82 ± 0.60	**	3.37 ± 0.23	***
Ref-2	Apollo Beach	Reference	--	--	4.53 ± 0.35	***	1.54 ± 0.16	***	-2.99 ± 0.44	***	3.30 ± 0.34	***
Ref-3	Robinson Preserve	Reference	--	--	4.87 ± 0.23	***	3.90 ± 0.21	***	-0.98 ± 0.31	**	3.03 ± 0.37	***
Ref-4	South Cockroach Bay	Reference	--	--	5.57 ± 0.31	***	3.52 ± 0.20	***	-2.05 ± 0.38	***	2.56 ± 0.27	***
Ref-5	Bullfrog Creek	Reference	--	--	7.40 ± 0.33	***	7.19 ± 0.22	***	-0.20 ± 0.41	ns	9.10 ± 0.24	***
Ref-6	Little Cockroach Bay	Reference	--	--	3.67 ± 0.27	***	2.23 ± 0.18	***	-1.44 ± 0.35	***	1.16 ± 0.26	***
Ref-7	Emerson Point	Reference	--	--	5.04 ± 0.23	***	3.26 ± 0.14	***	-1.78 ± 0.28	***	3.07 ± 0.24	***
Ref-8	East Cockroach Bay	Reference	--	--	9.11 ± 0.44	***	5.32 ± 0.31	***	-3.79 ± 0.54	***	3.76 ± 0.23	***
Ref-9	Wolf Branch	Reference	--	--	7.80 ± 0.73	***	5.02 ± 0.28	***	-2.78 ± 0.88	**	4.45 ± 0.25	***

ns = Not significantly different from 0 at the 0.05 level, * Significant at 0.01-0.05 level, ** Significant at the 0.01-0.001 level, *** Significant at < 0.001 level. [a] Identical numbers identify created (Cr) and reference (Ref) site pairings, [b] Assessed over 8.2-8.7 m depth, [c] Assessed over 50 cm depth, [d] Positive values denote root zone expansion, negative values denote shallow subsidence (compaction).

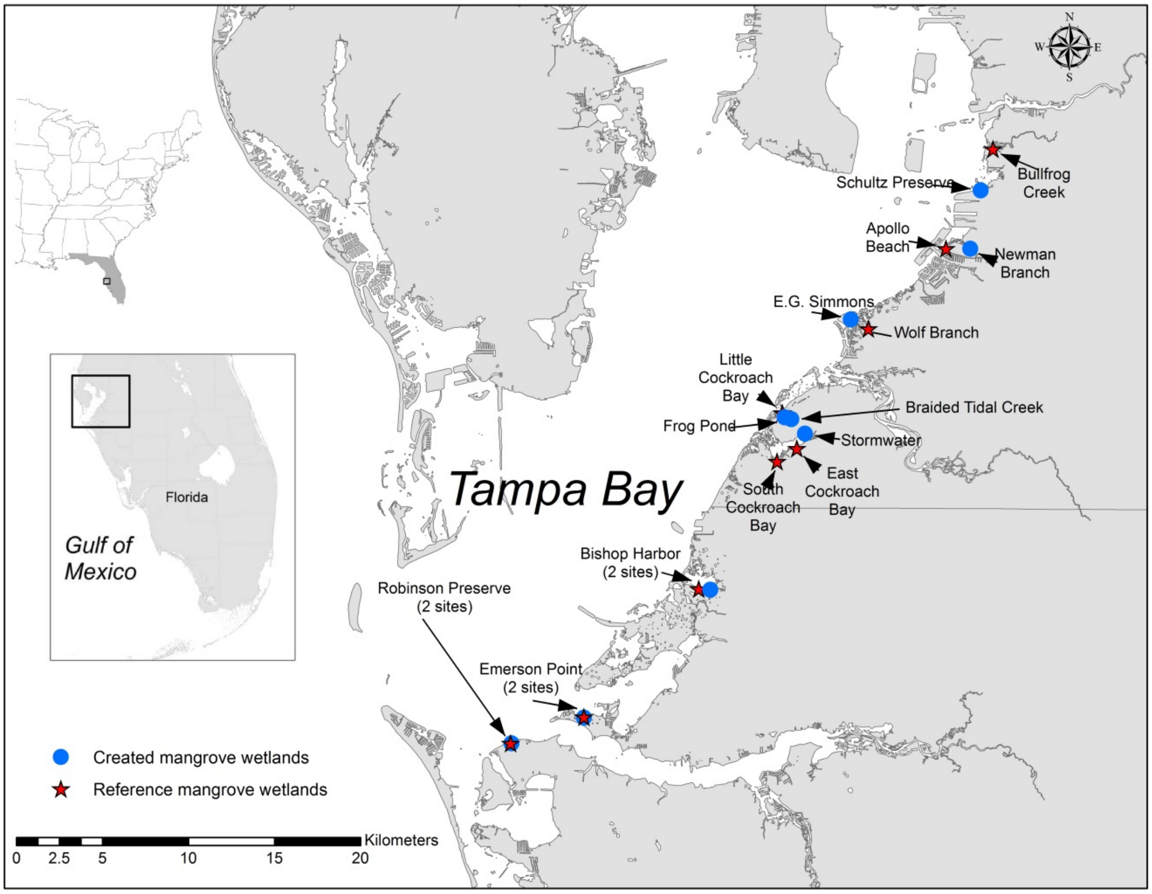

Supplementary Figure 1|Location of created mangrove wetlands and reference mangrove wetlands in Tampa Bay, Florida USA. Study sites included nine created mangrove wetlands positioned alongside of nine natural reference mangrove wetlands within 0.1-2.5 km of their corresponding created wetland in Tampa Bay, Florida, USA. (Map was created using ArcGIS v. 10.3 software by Esri, Redlands, California, USA. ArcGIS and ArcMap are the intellectual property of Esri and are used herein under license. The county shapefile was acquired from the Florida Geographic Data Library (FGDL), created by the University of Florida GeoPlan Center, and is in the public domain, http://www.fgdl.org)

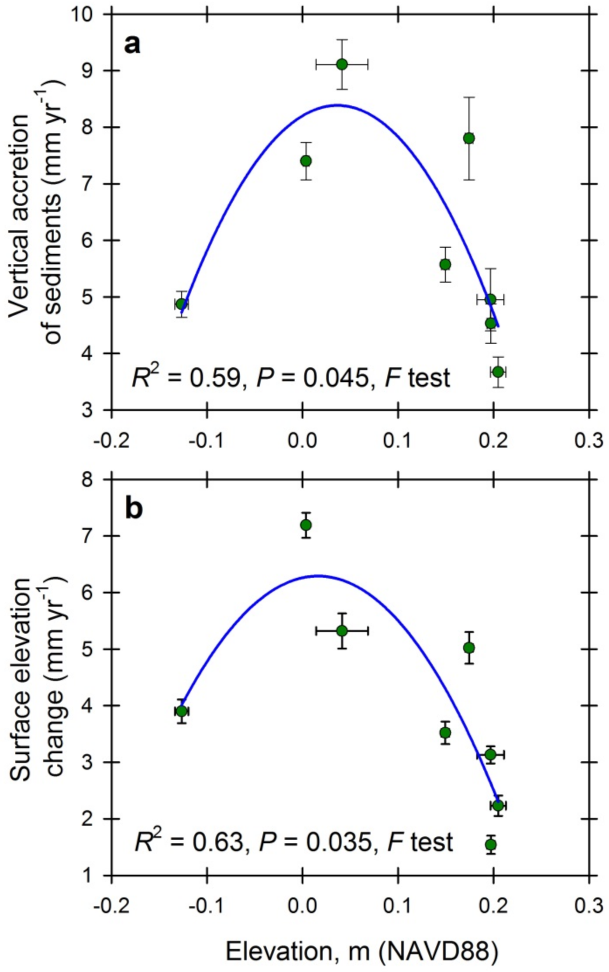

Supplementary Figure 2|Site elevation (NAVD88) versus vertical accretion of sediments and surface elevation change used to initialize an empirical sea-level rise response model. Relationships between site elevation (NAVD88) and either vertical accretion of sediments (**a**) or surface elevation change (**b**) were developed for naturally established reference mangrove wetlands within 0.1-2.5 km of their corresponding created tidal wetland in Tampa Bay, Florida, USA. One reference wetland was omitted because of inaccurate GPS survey data (n=8). Symbols represent means (±SE, bi-directional) of average vertical accretion of sediments or surface elevation change response by site. Solid blue lines are polynomial regressions: **a**, vertical accretion of sediments = 8.2021 + 10.0599(x) – 137.7121(x^2); **b**, surface elevation change = 6.2619 + 3.6304(x) – 112.0401(x^2); where x is site elevation in meters, NAVD88.
